# Analysis of association between common variants of uncoupling proteins genes and diabetic retinopathy in a Chinese population

**DOI:** 10.1186/s12881-020-0956-y

**Published:** 2020-02-06

**Authors:** Peiyao Jin, Zhiqiang Li, Xian Xu, Jiangnan He, Jianhua Chen, Xun Xu, Xuan Du, Xuelin Bai, Bo Zhang, Xiangui He, Lina Lu, Jianfeng Zhu, Yongyong Shi, Haidong Zou

**Affiliations:** 1 Department of Ophthalmology, Shanghai General Hospital, Shanghai Jiao Tong University School of Medicine (originally named“Shanghai First People’s Hospital”), Shanghai Key Laboratory of Ocular Fundus Diseases, Shanghai Engineering Center for Visual Science and Photomedicine, Shanghai Engineering Center for Precise Diagnosis and Treatment of Eye Diseases, National Clinical Research Center for Eye Diseases, No.100 Haining Road, Shanghai, 20080 China; 2 Bio-X Institutes, Key Laboratory for the Genetics of Developmental and Neuropsychiatric Disorders (Ministry of Education), Shanghai Jiao Tong University, Shanghai, 200030 China; 3 Shanghai Eye Disease Prevention and Treatment Center, Shanghai Eye Hospital, Shanghai, 200040 China; 4 Xinjing Community Health Service Centre, Shanghai, 200335 China

**Keywords:** Diabetic retinopathy, Uncoupling proteins, Single nucleotide polymorphisms, Type 2 diabetes

## Abstract

**Background:**

The aim of this study was to explore the association between diabetic retinopathy (DR) and the variants of uncoupling proteins (UCPs) genes in a Chinese population of type 2 diabetes, in total and in patients of different glycemic status separately.

**Methods:**

This case-control study included a total of 3107 participants from two datasets, among which 662 were DR patients (21.31%). Eighteen tag single nucleotide polymorphisms (SNPs) of *UCP1*, *UCP2*, and *UCP3* were selected as genetic markers. TaqMan probes, Sequenom MassARRAY MALDI-TOF mass spectrometry platform and Affymetrix Genome-Wide Human SNP Array were used for genotyping. Online SHEsis software was used for association analysis. Bonferroni correction was used for multiple comparisons correction.

**Results:**

Three SNPs of *UCP1*: rs7688743 (A allele, OR = 1.192, *p* = 0.013), rs3811787 (T allele, OR = 0.863, *p* = 0.023), and rs10011540 (G allele, OR = 1.368, *p* = 0.004) showed association with DR after the adjustment of glucose, but only rs10011540 was marginally significantly associated with DR when Bonferroni correction was strictly applied (p_adj_ = 0.048). In patients with uncontrolled glucose, rs7688743 (A allele, *p* = 0.012, OR = 1.309), rs10011540 (G allele, *p* = 0.033, OR = 1.432), and rs3811787 (T allele, *p* = 0.022, OR = 0.811) were associated with DR, while in participants with well controlled glucose, the rs2734827 of *UCP3* was associated with DR (A allele, *p* = 0.017, OR = 0.532). Rs3811787 of *UCP1* showed a protective effect to sight threatening DR (T allele, *p* = 0.007, OR = 0.490), and the association existed after the adjustment for environmental factors and the correction. In patients with uncontrolled glucose, the rs3811787 of *UCP1* (T allele, *p* = 0.017, OR = 0.467) and the rs591758 of *UCP3* (C allele, *p* = 0.026, OR = 0.103) were associated with STDR. While in those with well controlled glucose, only the rs7688743 of *UCP1* showed a protective effect (A allele, *p* = 0.024, OR = 0.049). None of the associations remain significant when Bonferroni correction was strictly applied (all *p* < 0.05).

**Conclusions:**

The rs10011540 and rs3811787 of the *UCP*1 gene was marginally significantly associated with DR in Chinese type 2 diabetes patients. There might be different mechanisms of DR development in patients with different glycemic status.

## Background

The age-standardized prevalence of diabetes in Chinese adult population reached 11.6% in 2013, and the number is still climbing [1]. Diabetic retinopathy (DR) is a common micro-vascular complication of diabetes, and the prevalence of DR is 25–43.1% in Chinese diabetic population [2–4]. DR is the main cause of blindness among people in productive age in several developed countries [5, 6]. The high prevalence of diabetes in China makes DR a big threat of vision loss in Chinese population.

DR is a multifactorial disease associated with both genetic and environmental factors [7, 8]. It is widely accepted that high glucose, high blood pressure and long diabetes duration are the most important environmental risk factors of DR progression [9]. Since 2003, our study group has been helping diabetes patients to control hyperglycemia and hypertension in order to prevent and control DR. However, DR still progressed in a number of patients with well controlled glucose and blood pressure [10]. This might be related to the “metabolic memory” phenomenon, which means the damages caused to cells and tissues by previous hyperglycemia persists after the normalization of glucose levels [11]. The mechanism of metabolic memory is not fully understood, but the genetic factors might be involved, because not all of the diabetes patients with hyperglycemic history develops metabolic memory.

Oxidative stress induced by hyperglycemia not only works as a vital part in the development of DR, but also plays a key role in the pathogenesis of metabolic memory [12]. The overproduction of mitochondrial reactive oxygen species (ROS) activates several molecular and biochemical pathways, and leads to long-lasting damage to tissues even after the glycemic status was controlled [13]. Uncoupling proteins (UCPs) are a group of mitochondrial anion-carrier proteins that can reduce the formation of mitochondrial ROS through reducing the proton motive force across the mitochondrial inner membrane [14]. The protective effect of UCPs in diabetic complications and the association between DR and *UCPs* genes were reported by several studies [15–18]; however, such association was not always verified [19, 20].

Previous researches suggested that apart from different study design, ethnicity, and synergetic effects, the interaction between genes and nutrients could be a possible reason for the inconsistency among different studies [21]. Increased glucose leads to an elevated expression of *UCPs*, while elevated *UCP*2 expression leads to a decreased insulin secretion and increased glucose [22, 23]. Thus, the interaction between UCPs and glucose level might affect the association between *UCP*s and DR; however, no previous study has taken the glucose controlling status into consideration.

The present case-control study is aimed to explore the association between DR and the variants of *UCPs* genes in a Chinese type 2 diabetes population. The relation between DR and *UCPs* was investigated among all the participants, and in participants with different glycemic status separately.

## Methods

### Setting and participants

The present study included two datasets collected from two separate studies.

The first study (Dataset 1) was a community-based cross-sectional study conducted in Xinjing community, Shanghai, China. A total of 1872 Han Chinese diagnosed with type 2 diabetes, including 528 DR patients (28.21%), were included into the study. Their mean age was 67.88 ± 9.50 years, and their mean diabetes duration was 7.17 ± 6.49 years. seven hundred sixty-four of the participants were male (40.81%). All of the participants in the first study have glucose records of the last 3 years. They went through systematic and ophthalmic exams during a survey, and the severity of DR was classified in these participants.

The second study (Dataset 2) was a sub-study of a genome-wide association study (GWAS) [24]. Participants of dataset 2 were collected from clinic, and most of them were new diagnosed diabetes patients. A total of 1235 type 2 diabetes patients of Han Chinese were included in this study, among which 134 were diagnosed with DR (12.17%). Their mean age was 64.78 ± 9.49 years, and 482 of the participants are male (39.02%). Their diabetes duration were not collected, past glucose level not recorded, and the severity of DR not classified. The comprehensive systemic and ophthalmology exams included in dataset 1 were not provided for the participants in dataset 2.

The study population of the two studies were both Han Chinese collected from Shanghai, China. Participants in dataset 2 were younger compared with those in dataset 1 (*p* < 0.01), but no difference existed in gender proportion (*p* = 0.32). In dataset 2, participants with DR were older than the others (71.07 ± 7.46y vs 64.02 ± 9.42y, *p* < 0.01). Age and gender were not related with DR in dataset 1.

### Systemic and ophthalmology exams in the community-based study (dataset 1)

The Xinjing Community Health Service Center created a health information database covering almost all of the residents in 1995, and the database has been updated annually since then. Diabetic residents have their hemoglobin A1c (HbA1c) and blood glucose monitored once every 3 months, and the results were recorded in the database. According to the glucose goal recommended by the American Diabetes Association, those patients with HbA1c under 6.5% for 3 whole years were considered glucose well controlled [25]. On the other hand, patients who didn’t meet with this standard were considered glucose uncontrolled.

A comprehensive systemic and eye examinations were conducted in each of the participant. Height and weight were measured at the survey site, and body mass index (BMI) was calculated as weight (kg)/height (m)^2^. Systolic pressure ≥ 140 mmHg and/or diastolic pressure ≥ 90 mmHg was considered high blood pressure. Serum creatinine, total cholesterol and triglycerides were measured using an enzymatic assay. Serum triglyceride ≥1.7 mmol/L was considered hyperlipidemia, serum total cholesterol ≥5.2 mmol/L was considered hypercholesterolemia, and serum creatinine ≥104 umol/L means hypercreatininemia. HbA1c was measured by ion exchange chromatography. Patients’ eyelid, conjunctiva, cornea, iris, and lens were examined with a slit-lamp microscope (YZ-5, Liuliu Medical Instrument Company, Suzhou, China), and the vitreous and fundus were examined by a direct ophthalmoscope by ophthalmologists.

All of the participants were screened for DR using the standardized protocol described in our previous paper [4]. Once early DR was suspected, the patient received further exams later in our hospital, including optical coherence tomography and fundus fluorescein angiography, to confirm the diagnosis. Participants’ retina photographs were read by two ophthalmologists independently (k = 0.874). The classification of DR severity was based on the Early Treatment Diabetic Retinopathy Study (ETDRS) scale [26].

In the main analysis, patients diagnosed with any degree of DR were included into the case group, no matter of the DME degree. While participants with no DR were included into the control group, no matter of the DME degree. In the sub-group analysis, patients with sight threatening DR (STDR) were included into the case group, which includes severe non-proliferative DR (NPDR), proliferative DR (PDR) and clinically significant macular edema (CSME). While participants with no DR and no DME were included into the control group. Only the participants of dataset 1 were included into the sub-group analysis, because the severity of DR was not classified in dataset 2.

### Tag single nucleotide polymorphisms (SNPs) selection and genotyping

Genomic DNA was extracted from peripheral blood samples using Quick Gene DNA whole blood kit L (FUJIFILM) protocol. Eight tag SNPs of *UCP1*, 3 tag SNPs of *UCP2*, and 7 tag SNPs of *UCP3* were selected using Haploview software (version 4.2), with pair-wise tagging r^2^ ≥ 0.6 and minor allele frequency (MAF) ≥ 0.05 based on the HapMap CHB dataset. The SNPs reported to be associated with diabetes by previous study, including rs1800592, rs659366, and rs660339, were also included in this study. The coverage was 100% calculated by Haploview software. Details of all the 18 SNPs were listed in Additional file 1: Table S1.

For the first community-based study, the rs660339 of *UCP*2 was genotyped by TaqMan probes, and the rest of the selected SNPs were genotyped using the Sequenom MassARRAY matrix-assisted laser desorption ionization-time of flight (MALDI-TOF) mass spectrometry platform (Sequenom, San Diego, CA). MassARRAY design software package (v4.0) was used to design the specific SNP filtering. The quality of PCR products were ascertained before the genotyping. Shrimp alkaline phosphatase was used to dephosphorylate residual nucleotides before iPLEX Gold reaction. After the single-base extension, the products were desalted with spectro clean resin (Sequenom), and 10 nl of the final products were spotted onto the Spectro Chip using the MassARRAY Nano-dispenser. The MassARRAY Analyzer Compact MALDI-TOF mass spectrometer was used to determine product mass. An assay with a call rate of less than 95% within the same Spectro Chip was considered below standard.

For the second GWAS study, SNPs were genotyped using the Affymetrix Genome-Wide Human SNP Array 6.0, and imputed using IMPUTE2 based on the 1000 Genomes release 20,101,123 reference panel. Quality control was conducted as described in the previous paper [24]. The genotyping information of each SNP was indicated in Additional file 1: Table S1, and the info of all imputed SNPs were over 0.8.

### Statistical analysis

Online SHEsis software (http://shesisplus.bio-x.cn/SHEsis.html) was used to analyze the Hardy-Weinberg equilibrium (HWE), allele frequency, and association test for each SNP [27]. Bonferroni correction (based on the total number of markers tested) was used for multiple comparisons correction, and statistical significance was defined as p<0.05 (two tailed).

SAS (version 9.3, SAS Institute, Cary, NC, USA) was used for statistical analysis of the systemic and eye measurements. Continuous variables were presented as means ± standard deviation, and categorical data were presented as rates (proportions). The data distribution was examined by Kolmogorov-Smirnov Test. For normally distributed continuous variables, t test was used to compare the means between groups. For variables that doesn’t follow Gaussian distribution, a Kruskal-Wallis H test was used. The categorical variables were compared with the Chi-square test. Distribution of alleles and genotypes between the case and the control groups were compared with the Chi-square test. Multivariate logistic regression analysis was used to get the odds ratios (OR) and their 95% confidential intervals (CIs) of each SNP before and after adjusting for various clinical variables. The genetic models tested in the study included additive, recessive and dominant models. Meta-analysis was used to combine the results of dataset1 and dataset2, and Cochran’s Q test was used for the heterogeneity test.

## Results

The rs660339 of *UCP2* and the rs1626521 of *UCP3* were excluded from further analysis because of low call rates (≤95%). The rs632862 of *UCP2* was excluded for MAF < 0.01, the rs668514 of *UCP3* was excluded for unsuccessful genotyping, and the rs15763 of *UCP3* was excluded due to disagree with HWE test (Additional file 1: Table S1). Thus, a total of 13 SNPs were included into further analysis.

### The association between UCPs and DR in all participants

A total of 3107 type 2 diabetic participants were included in the whole study, including 1872 from dataset 1 and 1235 from dataset 2. Three SNPs of *UCP1*: rs7688743, rs10011540, and rs3811787, showed association with DR (Table 1). The A allele of rs7688743 (*p* = 0.013, OR = 1.192), and the G allele of rs10011540 (*p* = 0.004, OR = 1.368) were risk factors of DR, while the T allele of rs3811787 was less frequent in DR patients (*p* = 0.023, OR = 0.863). Only rs10011540 showed marginally significant association with DR when Bonferroni correction was strictly applied (p_adj_ = 0.048). No difference was observed in the association results between the two datasets (all Q > 0.05).

**Table 1:** The association between uncoupling proteins genes variants and diabetic retinopathy

Chr.^a^	rs ID	Position (hg19)	A1^b^	Dataset 1 (528 cases vs 1344 controls)		Dataset 2 (134 cases vs 1101 controls)		Meta-analysis			
				OR^c^ [95% CI^d^]	*P*	OR [95% CI]	*P*	OR	*P*	*P*_*adj*_^e^	Q^f^
4	rs6818140	141,487,955	G	1.028 [0.852–1.240]	0.772	1.190 [0.870–1.629]	0.277	1.068	0.421	1.000	0.4326
4	rs7688743	141,488,795	A	1.152 [0.982–1.351]	0.083	1.322 [0.999–1.750]	0.050	1.192	0.013	0.173	0.4028
**4**^g^	**rs10011540**	**141,489,996**	**G**	**1.312 [1.031–1.670]**	**0.027**	**1.571 [1.012–2.438]**	**0.043**	**1.368**	**0.004**	**0.048**	**0.4814**
4	rs3811787	141,490,419	T	0.861 [0.747–0.993]	0.039	0.870 [0.657–1.151]	0.328	0.863	0.023	0.293	0.9501
4	rs3811790	141,491,576	A	0.946 [0.816–1.098]	0.466	0.938 [0.718–1.225]	0.638	0.944	0.387	1.000	0.9549
4	rs3811791	141,491,773	C	0.973 [0.823–1.150]	0.745	0.874 [0.642–1.190]	0.391	0.949	0.488	1.000	0.5499
4	rs1472268	141,492,943	A	1.007 [0.873–1.161]	0.926	0.991 [0.769–1.277]	0.943	1.003	0.961	1.000	0.9130
4	rs1800592	141,493,961	T	0.996 [0.864–1.148]	0.959	1.015 [0.787–1.308]	0.910	1.001	0.993	1.000	0.9013
11	rs659366	73,694,754	T	0.998 [0.862–1.156]	0.983	1.249 [0.965–1.617]	0.091	1.055	0.414	1.000	0.1394
11	rs591758	73,698,060	C	0.989 [0.854–1.145]	0.882	1.206 [0.932–1.561]	0.154	1.038	0.567	1.000	0.1896
11	rs1685356	73,712,859	T	1.053 [0.913–1.215]	0.477	0.910 [0.699–1.185]	0.485	1.019	0.770	1.000	0.3415
11	rs3741135	73,713,960	A	1.022 [0.882–1.184]	0.772	1.305 [1.003–1.698]	0.047	1.083	0.223	1.000	0.1123
11	rs2734827	73,716,277	A	0.990 [0.743–1.320]	0.947	0.868 [0.605–1.247]	0.444	0.941	0.597	1.000	0.5774

^a^ Chr., chromosome; ^b^ A1, effect allele; ^c^ OR, odds ratio; ^d^ CI, confidence Interval; ^e^ *P*_*adj*_, Bonferroni correction based on the total number of markers tested (*n* = 13); ^f^ Cochran’s Q statistic; ^g^ The SNP with corrected *P* < 0.05 in the meta-analysis was marked in bold

The association between the candidate SNPs genotypes and DR was presented in Table 2. The genotype of GG and GT of rs10011540 showed significantly association with DR in the additive model (GG vs GT vs TT: *p* = 0.004, p_adj_ = 0.048, OR = 1.373) and in the Dominant model (GG + GT vs TT: *p* = 0.003, p_adj_ = 0.039, OR = 1.410). Additionally, the genotype analysis of rs3811787 (*p* = 0.027, OR = 0.868), rs7688743 (*p* = 0.014, OR = 1.196) and rs3811790 (*p* = 0.037, OR = 0.828) all showed association with DR, but not significant after the Bonferroni correction. No positive associations between DR and SNPs was found in the recessive model. No other SNP genotypes was found to be associated with DR.

**Table 2:** The association of uncoupling proteins genotypes and diabetic retinopathy

rs ID	Genotype	Dataset 1				Dataset 2				Meta-analysis			
		DR	NDR	OR^a^ [95% CI^b^]	P	DR	NDR	OR [95% CI]	P	OR	*P*	*P*_*adj*_^c^	Q ^d^
**rs10011540**^e^	**GG**	**4**	**7**	**Add**^f^**:1.312 [1.026–1.676]**	**0.030**	**0**	**3**	**Add:1.607 [1.021–2.527]**	**0.040**	**1.373**	**0.004**	**0.048**	**0.440**
	**GT**	**101**	**204**	**Dom**^g^**:1.332 [1.027–1.726]**	**0.030**	**26**	**138**	**Dom:1.685 [1.066–2.664]**	**0.025**	**1.410**	**0.003**	**0.039**	**0.380**
	**TT**	**423**	**1132**			**100**	**914**						
rs3811787	TT	121	342	Add:0.867 [0.753–0.998]	0.047	23	238	Add:0.870 [0.657–1.151]	0.329	0.868	0.027	0.351	0.985
	GT	246	657	Dom:0.787 [0.630–0.983]	0.035	56	477	Dom:0.859 [0.553–1.334]	0.499	0.801	0.029	0.377	0.729
	GG	161	345			31	241						
rs7688743	AA	38	76	Add:1.151 [0.977–1.355]	0.092	9	57	Add:1.351 [1.009–1.808]	0.043	1.196	0.014	0.182	0.347
	AG	219	528	Dom:1.162 [0.950–1.422]	0.145	63	425	Dom:1.481 [1.027–2.136]		1.229	0.022	0.286	0.256
	GG	271	740			58	575						
rs3811790	AA	80	168	Add:0.952 [0.821–1.104]	0.517	17	133	Add:0.937 [0.715–1.227]	0.634	0.949	0.425	1	0.916
	AC	213	644	Dom:0.817 [0.666–1.001]	0.051	58	522	Dom:0.866 [0.603–1.243]	0.434	0.828	0.037	0.481	0.785
	CC	235	532			59	446						

^a^ *OR* odds ratio, ^b^ *CI* confidence interval, ^c^ *P*_*adj*_ Bonferroni correction based on the total number of markers tested (*n* = 13), ^d^ Cochran’s Q statistic ^e^ the SNP with corrected *P* < 0.05 in the meta-analysis was marked in bold; ^f^ Add, the additive model; ^g^ Dom, the dominant model

The interaction effect of the rs10011540 and environmental factors on DR was further tested in dataset 1 participants by logistic regression analysis (Table 3), which used DR status as the dependent variables, and the rs10011540 and environmental features as the independent variables. It was suggested that rs10011540 was associated with DR after the adjustment for age, gender, BMI, high blood pressure, hypercreatininemia, hypercholesterolemia, hyperlipidemia and HbA1c (*p* = 0.025, OR = 1.360). While diabetes duration might have interaction with rs10011540.

**Table 3:** The association of SNPs and diabetic retinopathy after adjustment for environmental factors

Characteristics Number	rs10011540 and DR		rs3811787 and STDR	
	OR [95% CI]	p	OR [95% CI]	p
unadjusted	1.312 [1.026–1.676]	0.030	0.715 [0.553–0.926]	0.011
age	1.312 [1.026–1.676]	0.030	0.713 [0.550–0.924]	0.011
age + gender	1.312 [1.026–1.676]	0.030	0.713 [0.550–0.924]	0.011
age + gender+BMI^a^	1.307 [1.018–1.677]	0.036	0.720 [0.553–0.936]	0.014
age + gender+BMI + HBP^b^	1.311 [1.011–1.700]	0.041	0.755 [0.574–0.992]	0.044
age + gender+BMI + HBP + hypercreatininemia	1.310 [1.011–1.699]	0.042	0.756 [0.575–0.994]	0.045
age + gender+BMI + HBP + hypercreatininemia +hypercholesterolemia	1.310 [1.011–1.699]	0.042	0.756 [0.575–0.994]	0.045
age + gender+BMI + HBP + hypercreatininemia +hypercholesterolemia+hyperlipidemia	1.310 [1.011–1.699]	0.042	0.756 [0.575–0.994]	0.045
age + gender+BMI + HBP + hypercreatininemia +hypercholesterolemia+hyperlipidemia+HbA1c^c^	1.360 [1.039–1.779]	0.025	0.732 [0.548–0.977]	0.034
age + gender+BMI + HBP + hypercreatininemia +hypercholesterolemia+hyperlipidemia+HbA1c + DD^d^	1.259 [0.941–1.686]	0.121	0.770 [0.565–1.049]	0.098

^a^*BMI* body mass index, ^b^*HBP* high blood pressure, ^c^*HbA1c* glycosylated haemoglobin, ^d^*DD* diabetes duration

### The association between UCPs and DR in participants with well controlled and uncontrolled glucose

There were 1016 participants with uncontrolled high glucose in dataset 1, among which 356 had DR (35.04%). DR patients had significantly higher glucose and longer diabetes duration compared with those with no DR, while no other difference was found (Table 4). DR was associated with rs7688743 (A allele, *p* = 0.012, OR = 1.309), rs10011540 (G allele, *p* = 0.033, OR = 1.432), and rs3811787 (T allele, *p* = 0.022, OR = 0.811). However, none of those association remains when Bonferroni correction is applied (all p_adj_ > 0.05) (Table 5). After adjusted by HbA1c level, rs7688743 (*p* = 0.009, OR = 1.332), rs10011540 (*p* = 0.003, OR = 1.458), and rs3811787 (*p* = 0.044, OR = 0.827) were still associated with DR.

**Table 4:** The association between environmental factors and diabetic retinopathy in participants with and without controlled glucose

	Diabetic retinopathy						Sight threatening diabetic retinopathy					
	Well controlled glucose			Uncontrolled glucose			Well controlled glucose			Uncontrolled glucose		
	Case	Control	p	Case	Control	p	Case	Control	p	Case	Control	p
n	172	684		356	660		23	657		104	611	
age (year)	67.04 ± 9.89	67.32 ± 9.34	0.73	67.97 ± 10.18	68.62 ± 9.13	0.32	71.87 ± 6.58	67.11 ± 9.22	0.01	69.58 ± 10.10	68.29 ± 9.08	0.19
male	66 (38.37)	254 (37.13)	0.76	159 (44.66)	285 (43.18)	0.65	8 (34.78)	243 (36.99)	0.83	51 (49.04)	264 (43.21)	0.27
diabetes duration	5.88 ± 6.00	4.45 ± 4.88	0.01	11.61 ± 7.06	7.95 ± 6.07	< 0.01	7.11 ± 7.63	4.11 ± 4.64	0.13	13.25 ± 7.29	7.72 ± 6.10	< 0.01
BMI^a^	24.62 ± 2.86	24.46 ± 3.08	0.25	24.88 ± 3.45	25.08 ± 3.38	0.6	24.65 ± 3.05	24.81 ± 3.16	0.82	24.25 ± 3.56	25.15 ± 3.27	0.01
high blood pressure	125 (72.67)	472 (69.01)	0.4	286 (80.34)	515 (78.03)	0.42	17 (77.27)	448 (75.17)	0.82	93 (89.42)	519 (84.94)	0.23
hypercreatininemia	8 (4.65)	37 (5.41)	0.69	28 (7.87)	33 (5.00)	0.07	0 (0.00)	34 (5.18)	0.26	11 (10.58)	28 (4.59)	0.01
hypercholesterolemia	68 (39.53)	295 (43.13)	0.39	167 (46.91)	277 (42.03)	0.14	9 (39.13)	288 (43.84)	0.65	48 (46.15)	261 (42.03)	0.52
hyperlipidemia	40 (23.26)	198 (28.95)	0.14	134 (37.64)	245 (37.18)	0.88	2 (8.7)	191 (29.07)	0.03	34 (32.69)	232 (38.03)	0.3
HbA1c^b^ (%)	5.93 ± 0.38	5.92 ± 0.38	0.89	8.27 ± 1.46	7.69 ± 1.08	< 0.01	6.06 ± 0.36	5.92 ± 0.37	0.08	8.44 ± 1.57	7.70 ± 1.09	< 0.01

^a^*BMI* body mass index, ^b^*HbA1c* hemoglobin A1c

**Table 5:** The association between uncoupling proteins and diabetic retinopathy in participants with and without controlled glucose

Chr.^a^	rs ID	Position (hg19)	A1^b^	Well controlled glucose (172 cases vs 684 controls)		Uncontrolled glucose (356 cases vs 660 controls)	
				OR^c^ [95% CI^d^]	P	OR [95% CI]	P
4	rs6818140	141,487,955	G	0.857 [0.617–1.190]	0.357	1.164 [0.908–1.493]	0.231
4	rs7688743	141,488,795	A	0.935 [0.709–1.232]	0.634	1.309 [1.061–1.615]	0.012
4	rs10011540	141,489,996	G	1.129 [0.745–1.711]	0.567	1.432 [1.045–1.963]	0.033
4	rs3811787	141,490,419	T	0.959 [0.760–1.210]	0.724	0.811 [0.677–0.971]	0.022
4	rs3811790	141,491,576	A	0.978 [0.766–1.250]	0.862	0.962 [0.795–1.163]	0.687
4	rs3811791	141,491,773	C	1.121 [0.855–1.470]	0.408	0.921 [0.742–1.143]	0.454
4	rs1472268	141,492,943	A	1.098 [0.870–1.387]	0.432	0.927 [0.774–1.112]	0.416
4	rs1800592	141,493,961	T	1.105 [0.874–1.396]	0.404	0.927 [0.774–1.112]	0.416
11	rs659366	73,694,754	T	1.065 [0.829–1.368]	0.624	0.936 [0.773–1.134]	0.500
11	rs591758	73,698,060	C	1.092 [0.851–1.400]	0.491	0.908 [0.750–1.099]	0.320
11	rs1685356	73,712,859	T	0.927 [0.731–1.176]	0.534	1.169 [0.972–1.406]	0.096
11	rs3741135	73,713,960	A	1.139 [0.888–1.461]	0.305	0.935 [0.772–1.131]	0.488
11	rs2734827	73,716,277	A	0.532 [0.286–0.989]	0.017	1.153 [0.753–1.765]	0.514

^a^ *Chr.* chromosome, ^b^ *A1* effect allele, ^c^ *OR* odds ratio, ^d^ *CI* confidence Interval

Among the 856 participants with well controlled low glucose in dataset 1, 172 had DR (20.09%). DR patients had longer diabetes duration compared with the control group, while no other difference was observed (Table 4). DR was associated with rs2734827 of *UCP*3 (A allele, *p* = 0.017, OR = 0.532), and the association still existed after adjusted by HbA1c level (*p* = 0.045, OR = 0.531). However, the association didn’t remain when Bonferroni correction is applied (all p_adj_ > 0.05) (Table 5). No association between *UCP1* and DR was discovered in this population.

### The association between UCPs and STDR

Among all the participants in dataset1, a total of 127 participants with STDR (case group) and 1268 DM patients with neither DR nor DME (control group) were included into the sub-group analysis. The case group included 29 patients with PDR, 41 with severe NPDR, and 82 with CSME in at least one eye (25 of the 82 patients also had PDR or severe NPDR). The STDR prevalence in patients with well controlled glucose is significantly lower than that with uncontrolled glucose (3.38% vs 14.54%, *p* < 0.01). In those with well controlled glucose, STDR patients were older and more often to have hyperlipidemia. While in those with uncontrolled glucose, STDR patients tend to have longer diabetes duration, lower BMI, higher HbA1, and more often with hypercreatininemia. (Table 4).

When all participants were included, rs3811787 of *UCP1* showed a protective effect to STDR (T allele, *p* = 0.007, OR = 0.490) (Table 6), and the association existed after the adjustment for age, gender, BMI, high blood pressure, hypercreatininemia, hypercholesterolemia, hyperlipidemia and HbA1c (T allele, *p* = 0.034, OR = 0.732) (Table 3). The protective effect of genotype TT and GT was shown in the additive model (TT vs GT vs GG: OR = 0.715, *p* = 0.011) and in the recessive model (TT vs GT + GG: OR = 0.565, *p* = 0.021), but not in the dominant model (Additional file 2: Table S2).

**Table 6:** The association between UCPs and sight threatening diabetic retinopathy in participants with and without controlled glucose

rs ID	A1^a^	All sub-group participants (127 cases vs 1268 controls)		Well controlled glucose (23 cases vs 657 controls)		Uncontrolled glucose (104 cases vs 611 controls)	
		OR^b^ [95% CI^c^]	P	OR [95% CI]	P	OR [95% CI]	P
rs6818140	G	2.773 [0.636–12.085]	0.174	11.773 [0.832–166.650]	0.068	1.610 [0.336–7.722]	0.552
rs7688743	A	0.241 [0.058–1.005]	0.051	0.049 [0.004–0.672]	0.024	0.425 [0.092–1.954]	0.272
rs10011540	G	2.612 [0.592–11.528]	0.205	18.758 [1.006–349.653]	0.050	1.448 [0.304–6.907]	0.642
rs3811787	T	0.490 [0.293–0.822]	0.007	0.505 [0.158–1.617]	0.250	0.467 [0.251–0.871]	0.017
rs3811790	A	1.111 [0.687–1.798]	0.667	1.919 [0.612–6.015]	0.264	1.125 [0.643–1.970]	0.679
rs3811791	C	0.689 [0.385–1.234]	0.211	0.704 [0.187–2.644]	0.603	0.608 [0.299–1.238]	0.170
rs1472268	A	0.906 [0.005–166.826]	0.970	0.001 [< 0.01- > 999]	0.982	> 999 [< 0.01- > 999]	0.959
rs1800592	T	1.282 [0.007–236.211]	0.926	> 999 [< 0.01- > 999]	0.981	< 0.01 [< 0.01- > 999]	0.959
rs659366	T	2.790 [0.533–14.599]	0.224	0.506 [0.066–3.899]	0.513	5.665 [0.812–39.498]	0.080
rs591758	C	0.222 [0.040–1.237]	0.086	1.244 [0.125–12.341]	0.852	0.103 [0.014–0.762]	0.026
rs1685356	T	1.004 [0.699–1.443]	0.981	0.592 [0.262–1.340]	0.209	1.203 [0.793–1.826]	0.384
rs3741135	A	1.565 [0.838–2.924]	0.160	1.182 [0.343–4.068]	0.791	1.770 [0.819–3.825]	0.146
rs2734827	A	0.881 [0.481–1.615]	0.683	0.381 [0.050–2.922]	0.354	1.217 [0.618–2.399]	0.570

^a^ *A1* effect allele, ^b^ *OR* odds ratio, ^c^ *CI* confidence Interval

In patients with uncontrolled glucose, the rs3811787 of *UCP1* (T allele, *p* = 0.017, OR = 0.467) and the rs591758 of *UCP3* (C allele, *p* = 0.026, OR = 0.103) were associated with STDR. While in those with well controlled glucose, only the rs7688743 of *UCP1* showed a protective effect (A allele, *p* = 0.024, OR = 0.049) (Table 6). None of the associations remain significant when Bonferroni correction was strictly applied (all *p* < 0.05).

## Discussion

UCPs are a group of anion-carrier proteins located in the inner mitochondrial membrane with similar structure and different tissue distributions. Their roles is to mediate regulated proton leaking across the inner mitochondrial membrane. The *UCP1* gene is located in the 4th chromosome, and it is mainly expressed in brown adipose tissue, but the protein and mRNA of *UCP1* were also detected in retinal cells. The *UCP2* and *UCP3* genes are located within 8 kb of each other in a gene cluster in the 11th chromosome. *UCP2* is widely distributed in the whole body, while *UCP3* is mainly expressed in the skeletal muscle [14].

UCPs are candidate genes for the pathology of hypertension, cardiac injuries, obesity, and diabetes. Its mechanism is adjusting energy homeostasis though thermogenesis and regulating ROS release [28]. The association between UCPs and hypertension was discovered in lab studies [29, 30], and was verified in Asian population [31, 32]. Molecular epidemiology studies found UCP2 polymorphism alters the risk of developing CAD in some populations [33, 34], while the relation between UCPs and obesity was widely recognized in several populations [35–37].

The association between the *UCPs* genes and diabetes has been investigated by many studies, and the most often studied SNPs are the rs1800592 (−3826A/G) of the *UCP1* gene, the rs659366 (−866G/A), rs660339 (Ala55Val), and Ins/Del of the *UCP2* gene, and the rs1800849 (−55C/T) of the *UCP3* gene [19, 20]. Lab and molecular epidemiology studies found that UCPs polymorphisms are associated with diabetic micro-vascular complications, including diabetic neuropathy [38, 39], nephropathy [40, 41], and DR [15–17]. However, there are some controversial findings about the association between UCPs variants and DR [19, 20].

The present study included 3107 Chinese type 2 diabetic patients of Han race, and the results suggested that the rs7688743, rs10011540, and rs3811787 of *UCP1* were associated with DR; however, only the rs10011540 (− 122 A/C) of *UCP1* was still marginally significantly associated with DR under Bonferroni correction, and the G allele was a risk factor of DR in additive model and in dominant model. The association between the rs10011540 and type 2 diabetes has been reported in two previous researches in Asian population. A study including1800 Asian Indians indicated that a haplotype, the -3826A/ -122C/−Met of *UCP1*, is a genetic risk for developing type 2 diabetes [42]. Another research of 570 Japanese subjects found that, the rs10011540 and Met229Leu polymorphisms of *UCP1* are in LD, and they are associated with type 2 diabetes [43]. So far, the association between rs10011540 and DR has not been reported.

The rs10011540 is a T/G polymorphism located in the 5′ UTR in the *UCP1* gene, and the mutation alters the consensus sequence of insulin response sequence (IRS). The mRNA and protein concentrations of *UCP1* were induced by insulin at physiological dose, thus this polymorphism may affect the affinity of transcriptional factors for the IRS of *UCP1*, result in impaired promoter activity, decreased *UCP1* protein producing, increased ROS production and energy storage. Mori et al. measured the transfecting activity of this polymorphism by using COS cells by transfecting promoter-reporter constructs with or without the polymorphism [43]. They found that the transcriptional activity of a fragment of the 5′ UTR of the mutant G allele decreased to approximately 40% compared with that of the corresponding fragment of the wild type T allele, which indicated the important role of this IRS in the transcription of *UCP1*. Moreover, Fukuyama et al. found that the G allele of rs10011540 may contribute to the accumulation of hepatic lipid content, and the development of insulin resistance [44].

The sub-group analysis of STDR showed that rs3811787 of UCP1 had a protective effect to STDR. Previous researches have reported that rs3811787 is associated with obesity, abdominal fat accumulation, and diabetic nephropathy [45–47]. Our study revealed its association with another diabetic microvascular complication-DR. Rs3811787 locates at the − 412 position upstream from the transcription start site, and is in moderate LD (r2 = 0.53) with rs1800592, which was associated with several diabetic complications. Former study indicated that rs3811787 is located adjacent to the binding site for the c-Rel transcription factor, this could be a possible operating mechanism.

When all the participants in dataset 1were included, the association between DR/ STDR and SNPs was affected by HbA1c level, which indicated the status of glucose control is a significant confounder. In uncontrolled glucose and well controlled glucose groups, the SNPs of UCPs were independently associated with DR/STDR after the adjustment of HbA1c. The difference between the results of the uncontrolled and the controlled glucose subgroups in the present study suggested that there might be different mechanisms in patients with different glucose control status during the development of DR/STDR; however, the result was limited by small sample size, because it is hard to collect participants with years of glucose records into the study. Although three SNPs of *UCP1* were associated with DR in participants of poor glucose control, and one SNP of UCP3 was found to be associated with DR in diabetes patients with well controlled hyperglycemia, the significance did not remain after Bonferroni correction was applied. A similar situation also happen to the association between SNPs and STDR in participants with and without controlled glucose. In patients with uncontrolled glucose, rs3811787 of UCP1 and rs591758 of UCP3 were associated with STDR, while in those with controlled glucose, the rs7688743 of UCP1 was related to STDR. Therefore, our study suggested that UCPs could be related to metabolic memory, and future studies should consider glucose status when exploring the association between *UCPs* genes and DR.

Small sample size, especially in the subgroups of participants with glucose history records, is the main shortage of the present study. Moreover, other diabetic microvascular complications such as nephropathy and neuropathy was not explored in our study. Our study group is going to conduct another survey in the same population to screen for DR in those without DR currently, and the main purpose is to confirm the association between the potential risk SNPs of *UCPs* genes found in the present study and DR development.

## Conclusions

In conclusion, the present study indicated that the rs10011540 of the *UCP*1 gene is marginally significantly associated with DR, and rs3811787 is marginally significantly associated with STDR in Chinese type 2 diabetes patients. There might be different mechanisms in patients with different glucose control status during the development of DR.

## Supplementary information

**Additional file 1: Table S1.** The characteristics of 18 single nucleotide polymorphisms of the uncoupling proteins genes.

**Additional file 2: Table S2.** The association of uncoupling proteins genotypes and sight threatening diabetic retinopathy.

## Data Availability

The datasets used and/or analysed during the current study are available from the corresponding author on reasonable request. Please contact Haidong Zou: Email: zouhaidong@hotmail.com; Phone number: 86–21–63240090-6833; Fax number: 86–21-33011075; Address: No.100 Haining Road, Shanghai, China 200080.

## Abbreviations

BMI: Body mass index
DR: Diabetic retinopathy
GWAS: Genome-wide association study
HbA1c: Hemoglobin A1c
MAF: Minor allele frequency
ROS: Reactive oxygen species
SNPs: Single nucleotide polymorphisms
UCPs: Uncoupling proteins
