# Supplementary material for: Analysis of association between common variants of uncoupling proteins genes and diabetic retinopathy in a Chinese population
Source: BMC Med Genet. 2020 Feb 6;21:25. doi: 10.1186/s12881-020-0956-y (PMC7006419; doi:10.1186/s12881-020-0956-y)
Supplement: Supplementary file 1 — Additional file 1: Table S1. The characteristics of 18 single nucleotide polymorphisms of the uncoupling proteins genes. [file 12881_2020_956_MOESM1_ESM.docx]

Additional file 1: **Table S1.** The characteristics of 18 single nucleotide polymorphisms of the uncoupling proteins genes

| Gene and locus | rs ID | Contig position | Base change | MAF^a^ | P for HWE^b^ | Call rate | Genotyped /  Imputed | info |
| --- | --- | --- | --- | --- | --- | --- | --- | --- |
| *UCP*1  4q28-q31 | RS7688743 | 140567641 | G>A | 0.2576 | 0.93 | 0.96 | Imputed | 0.973 |
|  | RS3811787 | 140569265 | G>T | 0.4617 | 0.38 | 0.96 | Imputed | 0.936 |
|  | RS1472268 | 140571789 | A>T | 0.4998 | 1.0 | 0.96 | Genotyped | 1 |
|  | RS10011540 | 140568842 | T>G | 0.0866 | 0.38 | 0.96 | Imputed | 0.942 |
|  | RS3811790 | 140570422 | C>A | 0.3602 | 0.67 | 0.96 | Genotyped | 1 |
|  | RS6818140 | 140566801 | A>G | 0.1731 | 0.15 | 0.96 | Genotyped | 1 |
|  | RS1800592 | 140572807 | G>A | 0.4991 | 1 | 0.96 | Imputed | 0.998 |
|  | RS3811791 | 140570619 | T>C | 0.2372 | 0.92 | 0.96 | Genotyped | 1 |
| *UCP*2  11q13 | RS660339 | 73978059 | C>T | 0.4366 | 0.64 | 0.95 | Imputed | 0.961 |
|  | RS659366 | 73983709 | C>T | 0.4451 | 0.77 | 0.96 | Imputed | 0.97 |
|  | RS632862 | 73982376 | C>G | 0.0010 | 1.00 | 0.96 | Imputed | 0.959 |
| *UCP*3  11q13.4 | RS591758 | 73987015 | G>C | 0.4520 | 0.80 | 0.96 | Imputed | 0.976 |
|  | RS3741135 | 74002915 | C>T | 0.3280 | 0.28 | 0.96 | Imputed | 0.999 |
|  | RS668514 | 73995469 | C>T | 0.1264 | 1 | 0.96 | Imputed | 0.981 |
|  | RS2734827 | 74005232 | C>T | 0.1444 | 0.18 | 0.96 | Genotyped | 1 |
|  | RS1685356 | 74001814 | G>A | 0.3926 | 0.99 | 0.96 | Genotyped | 1 |
|  | RS15763 | 74000432 | C>T | 0.1942 | <0.01 | 0.96 | Imputed | 0.995 |
|  | RS1626521 | 74003294 | C>T | 0.1316 | 0.96 | 0.95 | Imputed | 0.996 |

^a^ MAF, minor allele frequency; ^b^ HWE, Hardy-Weinberg equilibrium
