# Supplementary material for: Analysis of association between common variants of uncoupling proteins genes and diabetic retinopathy in a Chinese population
Source: BMC Med Genet. 2020 Feb 6;21:25. doi: 10.1186/s12881-020-0956-y (PMC7006419; doi:10.1186/s12881-020-0956-y)
Supplement: Supplementary file 2 — Additional file 2: Table S2. The association of uncoupling proteins genotypes and sight threatening diabetic retinopathy. [file 12881_2020_956_MOESM2_ESM.docx]

Additional file 2: **Table S2.** The association of uncoupling proteins genotypes and sight threatening diabetic retinopathy

| **rs ID** | **Genotype** | **DR** | **NDR** | **OR^a^ [95% CI^b^]** | **P** | **P_adj_ ^c^** |
| --- | --- | --- | --- | --- | --- | --- |
| rs3811787 | TT | 21 | 329 | Add **^e^**:0.715[0.553-0.926] | 0.011 | 0.143 |
|  | GT | 63 | 608 | Dom **^f^**:0.690[0.468-1.017] | 0.061 | 0.793 |
|  | GG | 43 | 331 | Rec **^g^**:0.565[0.348-0.918] | 0.021 | 0.273 |

**^a^** OR, odds ratio; **^b^** CI, confidence interval; **^c^** P_adj_, Bonferroni correction based on the total number of markers tested (n = 13); **^e^** Add, the additive model; **^f^** Dom, the dominant model; **^g^** Rec, the recessive model
